# Ligand-induced sequestering of branchpoint sequence allows conditional control of splicing

**DOI:** 10.1186/1471-2199-9-23

**Published:** 2008-02-12

**Authors:** Dong-Suk Kim, Veronica Gusti, Kenneth J Dery, Rajesh K Gaur

**Affiliations:** 1 Division of Molecular Biology, Beckman Research Institute of the City of Hope, Duarte, CA 91010, USA; 2 MCDB Program, Iowa State University, Ames, IA 50011, USA; 3 Division of Immunology, Beckman Research Institute of the City of Hope, Duarte, CA 91010, USA; 4 Graduate School of Biological Sciences, Beckman Research Institute of the City of Hope, Duarte, CA 91010, USA

## Abstract

**Background:**

Despite tremendous progress in understanding the mechanisms of constitutive and alternative splicing, an important and widespread step along the gene expression pathway, our ability to deliberately regulate gene expression at this step remains rudimentary. The present study was performed to investigate whether a theophylline-dependent "splice switch" that sequesters the branchpoint sequence (BPS) within RNA-theophylline complex can regulate alternative splicing.

**Results:**

We constructed a series of pre-mRNAs in which the BPS was inserted within theophylline aptamer. We show that theophylline-induced sequestering of BPS inhibits pre-mRNA splicing both in vitro and in vivo in a dose-dependent manner. Several lines of evidence suggest that theophylline-dependent inhibition of splicing is highly specific, and thermodynamic stability of RNA-theophylline complex as well as the location of BPS within this complex affects the efficiency of splicing inhibition. Finally, we have constructed an alternative splicing model pre-mRNA substrate in which theophylline caused exon skipping both in vitro and in vivo, suggesting that a small molecule-RNA interaction can modulate alternative splicing.

**Conclusion:**

These findings provide the ability to control splicing pattern at will and should have important implications for basic, biotechnological, and biomedical research.

## Background

Pre-mRNA splicing is a fundamental process that joins exons by catalyzing the removal of intervening sequences (introns) from mRNA precursors. Pre-mRNAs are spliced in a two-step pathway catalyzed by the spliceosome, a dynamic macromolecular machinery consisting of five small nuclear ribonucleoproteins (U1, U2, U4, U5 and U6 snRNPs) and many non-snRNP proteins. In the first step, pre-mRNA is cleaved at the 5' splice site (ss) generating two splicing intermediates: a linear first exon, and an intron-second exon in a lariat configuration. In the second step, the 3'-hydroxyl group of the last nucleotide in first exon makes a nucleophillic attack at the phosphodiester bond separating the intron and the second exon (3' ss), enabling the joining of two exons and release of the intron as a lariat (for review, see references [1-3]).

The differential joining of 5' and 3' ss of a single pre-mRNA, a phenomenon known as alternative splicing, can generate variant mRNAs with diverse and often antagonistic functions [4-7]. Alternative splicing of pre-mRNA is now considered to be the most important source of protein diversity in vertebrates [8,9]. It is estimated that 35–60% of human genes generate transcripts that are alternatively spliced [10,11], and 70–90% of alternative splicing events affect coding capacity of genes [12,13]. Importantly, deregulated splice variant expression has been identified as the cause of a number of genetic disorders [14-16], and certain forms of cancer have been linked to unbalanced isoform expression from genes involved in cell cycle regulation or apoptosis [17-20]. Given the critical role of alternative splicing in a variety of cellular processes, strategies that could influence pre-mRNA splicing decisions will have far-reaching effects in biotechnology and medicine.

Initial efforts aimed at controlling pre-mRNA splicing exploited the intrinsic property of nucleic acids to bind specific pre-mRNA sequence and inhibit splicing [21]. However, susceptibility of antisense oligonucleotides to nuclease digestion, off-target effects, difficulty with delivery and localization led to the realization that improved methods are required [22]. Bifunctional molecules that function like an antisense oligonucleotide, but carry the binding site for a known splicing factor have proved to be useful in reprogramming pre-mRNA splicing [23-25]. While bifunctional molecules have overcome some of the problems of the original antisense-based approach, requirements of chemical modifications limit their utility. In addition, these approaches function in a constitutive manner, i.e., an antisense oligonucleotide directed to inhibit the splicing of a pre-mRNA will continue to do so as long as the oligonucleotide is available. Since alternative splicing of many pre-mRNAs is controlled by splicing regulators [6,26], a small molecule ligand that could mimic the function of a splicing regulator would be of broad general application in controlling gene expression [27].

We have previously shown that insertion of a theophylline-responsive riboswitch into the 3' ss region of a model pre-mRNA enables its splicing to be repressed during the second step specifically by theophylline [28]. Since the vast majority of human genes are alternatively spliced and splice site pairing in general occurs at early stages of the splicing pathway, we reasoned that sequestering of BPS in a theophylline aptamer would be a better approach for controlling alternative splicing. In this study, we tested this idea by constructing a series of pre-mRNAs in which BPS was inserted within theophylline aptamer. We show that theophylline-induced sequestering of BPS is highly specific and inhibits pre-mRNA splicing both in vitro and in vivo in a dose dependent manner. Our results indicate that the thermodynamic stability of RNA-theophylline complex and the location of BPS within this complex affect the efficiency of splicing inhibition. Finally, we show that sequestering of BPS within RNA-theophylline complex can modulate alternative splicing both in vitro and in cultured cells.

## Results

### Insertion of BPS within theophylline-responsive riboswitch confers ligand dependent control of splicing

**Figure 1:**
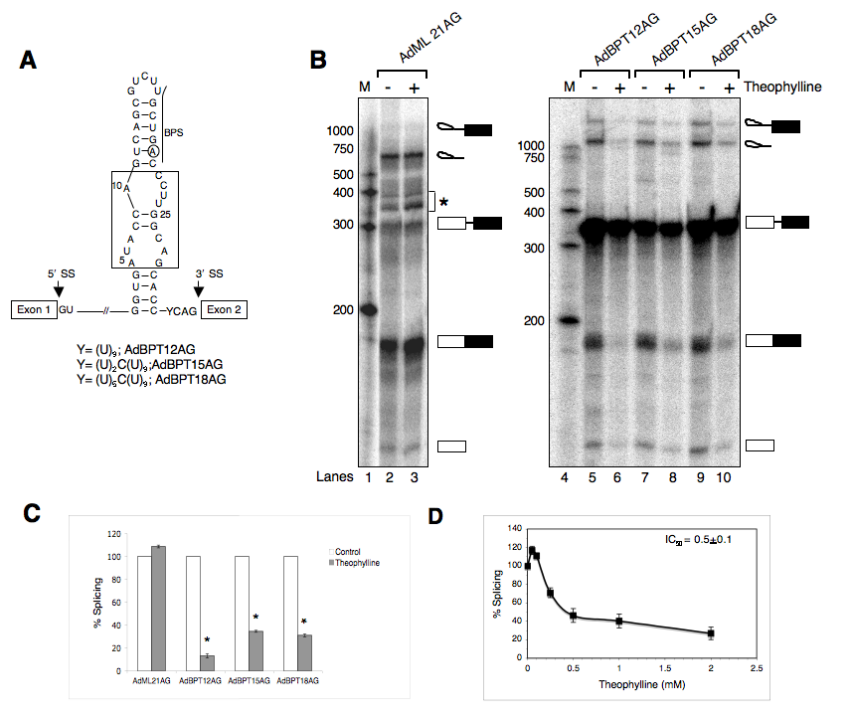
**Insertion of BPS within theophylline-responsive riboswitch confers ligand-dependent control of splicing**. (A) Schematic diagram of AdBPT pre-mRNAs. Underlined sequence and encircled adenosine residue represents the BPS and branch nucleotide, respectively. Open boxes represent exon sequences and horizontal lines between exons indicate introns. Arrow specifies 5' and 3' splice sites. The boxed residues within the secondary structure of theophylline binding aptamer are conserved for theophylline binding. The numbering system follows according to the reference [29]. (B) In vitro splicing of AdML 21AG and AdBPT pre-mRNAs. ^32^P-labeled pre-mRNAs were incubated in HeLa nuclear extracts at 30°C for 2 h in the absence or presence of theophylline (for experimental details, see methods section). Total RNA isolated from each reaction was fractionated on a 13% polyacrylamide denaturing gel. The bands corresponding to pre-mRNA substrates, intermediates and spliced products are indicated on the right. M, Century™-plus RNA size marker (Ambion). Asterisk (*) indicates degraded lariat. (C) Histogram depicting the effect of theophylline on the splicing of AdML 21AG and AdBPT pre-mRNAs. Splicing was calculated as the ratio of spliced mRNA to the total and normalized to the control (no theophylline). Data represent mean ± standard error of the mean (SEM) of three independent experiments. Asterisk represents significant change as compared to the control (*, *P *< 0.0005). (D) Theophylline inhibits the splicing of AdBPT15AG pre-mRNA in a dose-dependent manner. ^32^P-labeled AdBPT15AG pre-mRNA was incubated in HeLa nuclear extracts in the absence or with increasing concentration of theophylline for 2 h as described in (B). % Splicing calculated as described above and plotted against theophylline (mM). The values are expressed as mean ± SEM.

To investigate whether theophylline-induced sequestering of BPS would allow conditional control of splicing, we constructed a series of AdML pre-mRNA derivatives in which BPS was embedded within high-affinity theophylline binding aptamer (TBA) (Fig. 1A and see Additional File 1). These pre-mRNAs differ in terms of the distance of aptamer sequence from the 3' ss AG. ^32^P-labeled pre-mRNAs were transcribed as runoff transcripts using T7 RNA polymerase and gel purified RNAs were incubated in HeLa nuclear extract under the conditions that support in vitro splicing. The results presented in Fig. 1B demonstrate that these substrates underwent normal splicing, albeit with lower efficiency compared with the parent pre-mRNA (Fig. 1B compare lane 2 with lanes 5, 7 and 9). Significantly, splicing reactions performed in the presence of theophylline gave rise to lower yields of spliced mRNA, suggesting that theophylline-mediated sequestering of branchpoint inhibits pre-mRNA splicing (Fig. 1B, compare lanes 5, 7 and 9 with lanes 6, 8 and 10, respectively). Quantitation of these data indicate that theophylline inhibited the splicing of AdBPT pre-mRNAs by ~65–90%, but had no effect on the splicing of AdML 21AG, a pre-mRNA that does not contain theophylline-binding aptamer (Fig. 1C). We chose AdBPT15AG for further experiments because of its better splicing efficiency in the absence of theophylline; while 17% of AdBPT15AG pre-mRNA was converted into mRNA, only ~7% and 9% of AdBPT12AG and AdBPT18AG pre-mRNAs gave rise to spliced product, respectively.

To determine theophylline concentration that may be optimum for inhibiting pre-mRNA splicing, ^32^P-labeled AdBPT15AG pre-mRNA was subjected to in vitro splicing in the presence of increasing concentrations of theophylline. Results shown in Fig. 1D indicate that theophylline inhibited the splicing of AdBPT15AG pre-mRNA in a dose-dependent manner with an IC_50 _value 0.5 ± 0.1 mM. A slight increase in the splicing efficiency at lower concentration of theophylline may be the RNA stabilization rather than inhibitory effect of the ligand.

### Splicing inhibition through theophylline-induced sequestering of BPS is specific

**Figure 2:**
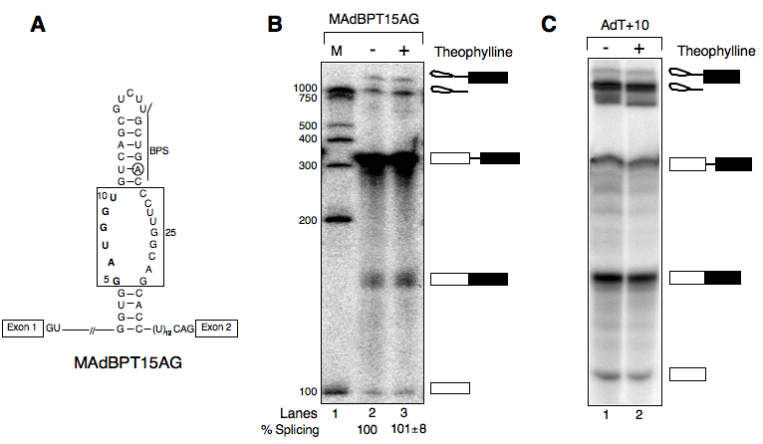
**Theophylline-dependent inhibition of pre-mRNA splicing is specific**. (A) The diagram of MAdBPT15AG pre-mRNA. The nucleotide residues shown in bold have been mutated to prevent the binding of theophylline. The RNA secondary structure is adapted from Zucker's M-FOLD program [62] (see Additional File 2). (B) Theophylline failed to inhibit the splicing of a substrate (MAdBPT15AG) that contains mutations within aptamer core. ^32^P-labeled MAdBPT15AG pre-mRNA was subjected to in vitro splicing in the absence (lane 2) or presence of theophylline (lanes 3) for 2 h. The extracted RNA was fractionated on a 13% polyacrylamide denaturing gel. The bands corresponding to splicing substrates, intermediates and spliced products are indicated on right. The % splicing calculated as in Fig. 1 and values are expressed as mean ± SEM. (C) AdT+10, a pre-mRNA in which theophylline-binding aptamer was inserted upstream of BPS was constructed as described in methods. ^32^P-labeled AdT+10 pre-mRNA was subjected to in vitro splicing in the absence (lane 1) or presence of theophylline (lane 2) for 2 h. The extracted RNA was fractionated on a 13% polyacrylamide denaturing gel (details as in Fig. 1B).

Our model of conditional control of splicing specifies that theophylline inhibits pre-mRNA splicing by binding to its cognate sequence, thereby sequestering the BPS within RNA-theophylline complex. To confirm that the splicing inhibition shown in Fig. 1B is specific, we performed the following experiments. First, we constructed MAdBPT15AG pre-mRNA that harbors mutations in the core of TBA, but otherwise is identical to AdBPT15AG (Fig. 2A, changed nucleotides shown in bold). On the basis of previously published reports [29,30], which suggest that the 15-nucleotide motif (Fig. 1A, residues enclosed in box) is essential for high-affinity theophylline binding, we predicted the splicing of MAdBPT15AG to remain unaltered in the presence of theophylline. Indeed, results shown in Fig. 2B demonstrate that theophylline failed to inhibit the splicing of MAdBPT15AG pre-mRNA. In contrast, a similar concentration of theophylline was found to inhibit the splicing of AdBPT15AG (compare lanes 7 and 8 in Fig. 1B with lanes 2 and 3 in Fig. 2B). Relatively low splicing efficiency of MAdBPT15AG pre-mRNA in the absence of theophylline suggests that mutations in the core of TBA although prevented theophylline binding, apparently this did not completely abolish secondary structure of the aptamer. Support for this interpretation comes from a web-based RNA-folding program indicating that the mutated aptamer can fold into a secondary structure (see Additional File 2).

Next, we generated AdT+10 pre-mRNA in which TBA was relocated upstream of BPS and analyzed its splicing in vitro using HeLa nuclear extract. As illustrated in Fig. 2C, this pre-mRNA underwent normal splicing in the presence of theophylline, suggesting that sequestering of BPS within RNA-theophylline complex is necessary for splicing inhibition. Finally, we examined the splicing of AdBPT15AG pre-mRNA in the presence of caffeine, a purine that is similar in shape and size to theophylline. Consistent with our previously published observation [28], caffeine failed to exert any influence on the splicing of AdBPT15AG pre-mRNA (data not shown). Taken together, we conclude that theophylline-dependent inhibition of pre-mRNA splicing is highly specific.

### Insertion of BPS in theophylline-binding aptamer does not activate cryptic branchpoint

Previous studies have shown that cryptic branchpoint activation allows accurate in vitro splicing of mammalian pre-mRNAs albeit with lower efficiency [31-33]. To determine if relatively lower splicing efficiency of AdBPT15AG pre-mRNA is due to the activation of a cryptic branchpoint, we mapped the position of the RNA branch. Preparative amounts of lariat intermediates were isolated from in vitro splicing reaction of AdBPT15AG pre-mRNA performed in the absence or presence of theophylline. The branchpoint was mapped by the primer extension of untreated or S100 treated lariat intermediate, which allows debranching of the lariat RNA [34]. The position of reverse transcriptase stop site was assigned by using a dideoxy-sequencing ladder from the parental DNA.

**Figure 3:**
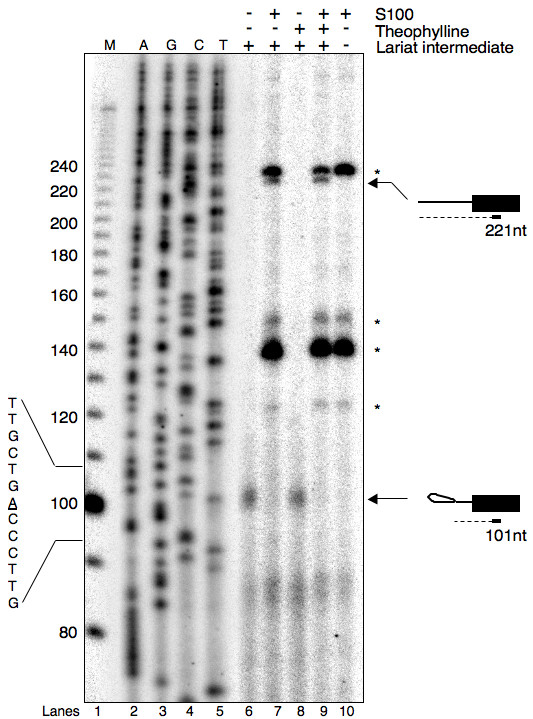
**Insertion of BPS in theophylline-binding aptamer does not activate cryptic branchpoint**. Lariat intermediate isolated from preparative splicing reactions of AdBPT15AG pre-mRNA performed in the absence (lanes 6 and 7) or presence of theophylline (lanes 8 and 9) was subjected to branchpoint mapping. Primer extension was performed on intact (lane 6, -S100) or debranched lariat intermediate (lanes 7 and 9, +S100). Asterisks (*) represent primer extension products from S100 total RNA (lane 10). The primer extension products were separated on a 13% denaturing polyacrylamide gel. The dideoxy-sequencing ladder from parental DNA is shown in lanes 2–5. M, 10 bp DNA ladder (Invitrogen).

The primer extension analysis of AdBPT15AG pre-mRNA derived lariat intermediate showed a single stop immediately before the branchpoint adenosine (Fig. 3, 101 nt product in lane 6). This band was not detected with unspliced pre-mRNA (data not shown) or the lariat intermediate that was treated with S100 extract (Fig. 3, lane 7). In contrast, the primer extension of S100 treated lariat species generated a 221 nt band corresponding to the size of linear intron-Exon 2 RNA (Fig. 3, compare lane 6 and 7). Importantly, the lariat intermediate isolated from theophylline treated splicing reaction gave rise to a single reverse transcriptase stop signal corresponding to 101 nt (Fig. 3, compare lane 6 with 8). In addition, this lariat intermediate was S100 sensitive, and the primer extension of debranched lariat intermediate produced a 221 nt species (Fig. 3, lane 8 and 9). The bands marked with asterisk are the primer extension products of S100 RNA likely generated due to the non-specific binding of the primer. This interpretation is supported by the fact that primer extension of RNA isolated from S100 extract resulted in identical reverse transcriptase stops (Fig. 3, compare lanes 7 and 9 with lane 10). We conclude that theophylline-dependent sequestering of BPS does not activate cryptic branchpoint.

### Thermodynamic stability of lower theophylline aptamer stem and location of BPS affects splicing inhibition

**Figure 4:**
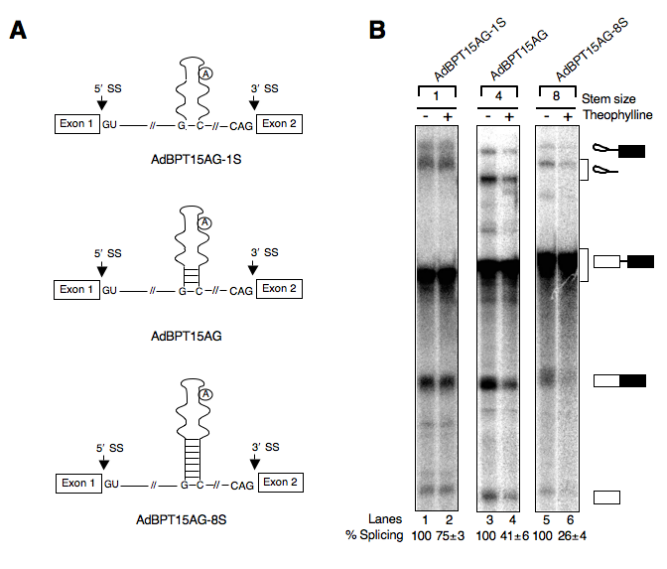
**Thermodynamic stability of lower theophylline aptamer stem affects splicing inhibition**. (A) Schematic diagram of AdBPT pre-mRNAs exhibiting varying length of lower aptamer stem. The encircled adenosine residue represents branch nucleotide. The distorted hairpin loop structure is the simplified secondary structure of theophylline aptamer. The horizontal lines in aptamer lower stem represent hydrogen bonds between complementary bases. (B) The splicing of AdBPT15AG pre-mRNA with lower aptamer stem of 4 bp (shown in Fig. 1A) was compared with substrates in which the stem was either decreased to a single bp (AdBPT15AG-1S) or increased to eight bp (AdBPT15AG-8S). ^32^P-labeled pre-mRNAs were subjected to in vitro splicing for 2 h in the absence (lanes 1, 3 and 5) or presence of theophylline (lanes 2, 4 and 6) as described in Fig. 1. The extracted RNAs were fractionated on a 13% denaturing polyacrylamide gel. The % splicing calculated as in Fig. 1 and values are expressed as mean ± SEM.

Biochemical and structural studies showed that the lower theophylline aptamer stem is not critical for ligand binding [29,35], but affects the stability of RNA-theophylline complex. If true, then altering the length of lower theophylline aptamer stem is expected to affect splicing suppression. To test this idea, we constructed two AdBPT15AG derivatives in which the length of lower theophylline aptamer stem was either decreased to a single bp (AdBPT15AG-1S) or increased to eight bp (AdBPT15AG-8S) (Fig. 4A). To assess the consequences of varied lower theophylline aptamer stem on pre-mRNA splicing, ^32^P-labeled substrates were incubated in HeLa nuclear extract in the absence or presence of theophylline under splicing conditions. We found that compared to ~60% splicing repression with AdBP15AG substrate, theophylline inhibited the splicing of AdBPT15AG-8S and AdBPT15AG-1S pre-mRNAs by ~75% and 25%, respectively (Fig. 4B, compare lanes 1, 3 and 5 with lanes 2, 4 and 6, respectively). These results indicate a direct correlation between the thermodynamic stability of lower theophylline aptamer stem and efficiency of splicing repression. It is worth mentioning here that although increasing the length of the lower aptamer stem resulted in stronger inhibition of splicing, stem >10 bp could inhibit splicing even in the absence of theophylline (Kim and Gaur, unpublished results).

**Figure 5:**
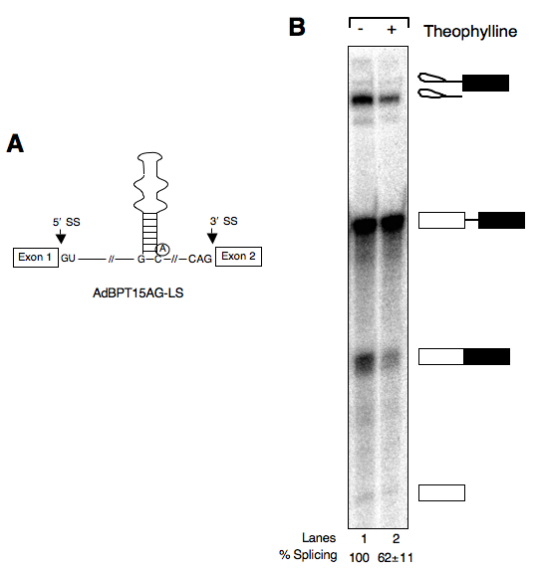
**The location of branchpoint sequence affects efficiency of splicing inhibition**. (A) Schematic representation of AdBPT15AG-LS pre-mRNA. The branchpoint sequence is present in the lower aptamer stem and the length of stem is 8 bp. (B) ^32^P-labeled AdBPT15AG-LS pre-mRNA was incubated in HeLa nuclear extract for 2 h in the absence (lanes 1) or presence of theophylline (lanes 2) as described in Fig. 1. The extracted RNA was fractionated on a 13% polyacrylamide denaturing gel. Percent splicing calculated as in Fig. 1 and values are expressed as mean ± SEM.

To examine whether the location of BPS within the aptamer affects efficiency of splicing inhibition, we constructed a model pre-mRNA in which BPS was inserted in the lower aptamer stem (Fig. 5A, AdBPT15AG-LS). Results presented in Fig. 5B demonstrate that relocation of BPS to the lower stem rendered host pre-mRNA less responsive to theophylline-mediated inhibition of splicing (compare lanes 5 and 6 in Fig. 4B with lanes 1 and 2 in Fig. 5B). Quantitation of the data indicate that compared to ~75% splicing inhibition with AdBPT15AG-8S, theophylline inhibited AdBPT15AG-LS pre-mRNA splicing by ~40%. The efficiency of splicing repression by theophylline is not significantly different between AdBP15AG and AdBPT15AG-LS pre-mRNAs, even though the latter contains a thermodynamically more stable lower stem (compare lanes 3 and 4 in Fig. 4B with lanes 1 and 2 in Fig. 5B). Based on these results, we propose a direct correlation between the thermodynamic stability of lower theophylline aptamer stem and efficiency of repression, provided the branchpoint sequence is present in the upper stem.

### Theophylline-dependent control of alternative splicing in vitro

**Figure 6:**
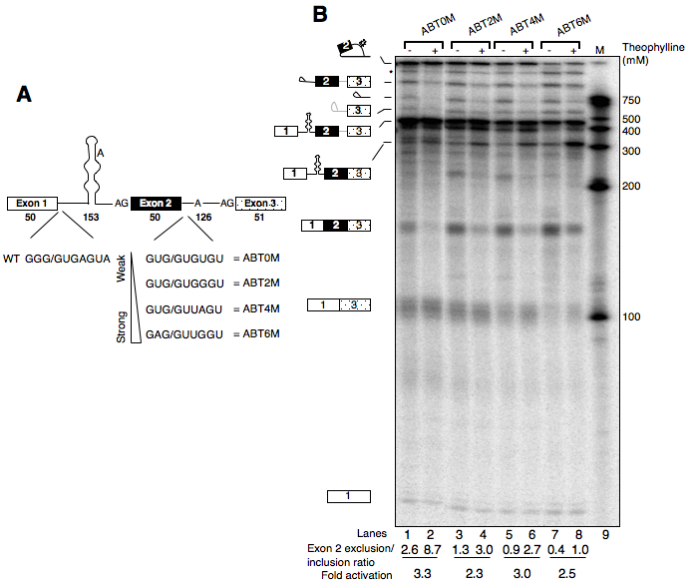
**Theophylline can modulate alternative splicing in vitro**. (A) Schematic representation of three-exon pre-mRNA substrates with varying strength of exon 2 5' ss. The sequence of first two exons and the entire intron 1 is identical to AdBPT15AG-8S. The sequence downstream of intron 2 5' ss, including BPS and exon 3 is from pRG1 [44]. The relative strength of exon 2 5' ss described here was based on the published report [45]. (B) Splicing in vitro of the panel of substrates (shown in A) displaying the effect of theophylline on the alternative splicing of exon 2. ^32^P-labeled AdML0M-6M pre-mRNAs were subjected to in vitro splicing in the absence (lanes 1, 3, 5 and 7) or presence of theophylline (lanes 2, 4, 6 and 8) as described in Fig. 1. The extracted RNA was fractionated on a 13% polyacrylamide denaturing gel. Other labeling is as in Fig. 1. The internal exon 2 exclusion: inclusion ratio for each substrate in the absence or presence of theophylline is shown below each lane.

Naturally occurring RNA structure elements [36-39] as well as artificial stem-loop structures [28,40-43] are known to influence splice site choice. To determine whether theophylline-induced secondary structure could influence alternative splicing, we constructed a series of pre-mRNAs comprising of three exons interrupted by two introns (Fig. 6A and Additional File 1). The sequence encompassing exon 1, intron 1 and exon 2 is identical to AdBPT15AG-8S, whereas sequence downstream of exon 2 5' ss is from pRG1, an adenovirus major late pre-mRNA derivative [44] (Fig. 6A). In addition, these pre-mRNAs differ from each other in terms of the strength of exon 2 5' ss, which increases in the order: ABT0M< ABT2M< ABT4M< ABT6M [45]. We hypothesized that sequestering of intron 1 branchpoint by theophylline would allow intron 2 branchpoint to choose between the 5' ss of exon 1 and 2 for the first step of splicing. Thus, depending upon which of the two 5' ss is utilized will determine the amount of exon 2 included/excluded mRNA.

To test this hypothesis, radioactively labeled ABT0M-6M pre-mRNAs were transcribed and incubated in HeLa nuclear extract in the absence or presence of theophylline as described earlier. Splicing of ABT0M substrate gave rise to two spliced products (also see below), a slower migrating band of ~150 nt and an additional band of ~100 nt (Fig. 6B, lane 1). To determine the identity of these mRNAs, representative bands were excised and RNA was subjected to RT-PCR followed by DNA sequencing. The sequencing results (not shown) indicated that the slower migrating band represents full-length mRNA, whereas faster moving band represents exon 2-skipped mRNA generated as a result of alternative splicing. Notably, theophylline shifted the splicing of ABT0M pre-mRNA in favor of exon 2 excluded isoform by repressing the activation of intron 1 branchpoint. This is evident by a sharp decline in the amount of full-length mRNA and concomitant increase of intron 1-retained mRNA (Fig. 6B, compare ~304 nt band and full length mRNA in lane 1 and 2). Further examination of these results across ABT0M to ABT6M panel indicate theophylline-dependent increase in the amount of intron 1-retained mRNA, and progressively weak suppression of exon 2-included mRNA (Fig. 6B, note the amounts of these mRNAs from left to right in theophylline treated lanes). While an increase in the amount of intron 1-containing mRNA is due to the inhibition of intron 1 excision, increasingly weak suppression of exon 2 included mRNA is the result of a gradual increase in the strength of exon 2 5' ss, which promotes exon 2 inclusion. These data indicate 2–3 fold increase in exon 2 excluded: included mRNA ratio across the panel (Fig. 6B). We also observed the presence of intron 1 containing mRNA in reactions where theophylline was excluded. This could simply be the result of lower efficiency of intron 1 excision, which was also observed in case of constitutively spliced substrate (Fig. 4B).

### Theophylline-dependent control of alternative splicing in vivo

**Figure 7:**
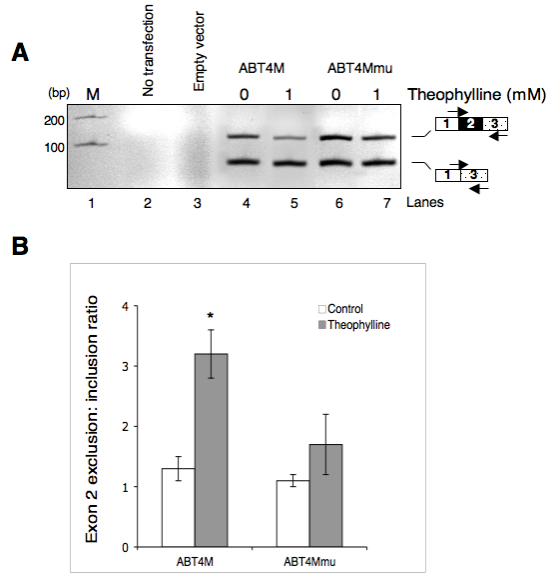
**Theophylline-mediated modulation of alternative splicing in vivo**. (A) HeLa cells were transiently transfected with empty vector, pcABT4M or pcABT4Mmu (containing mutations within core TBA). Cells were treated with buffer (lanes 4 and 6) or with 1 mM theophylline (lanes 5 and 7). The product of RT-PCR assays of total RNA isolated from each well was analyzed on a 2.5% agarose gel to estimate the effect of theophylline on the efficiency of exon 2 alternative splicing. The PCR amplified bands corresponding to exon 2 included or excluded mRNA is indicated. (B) The internal exon 2 exclusion: inclusion ratio for ABT4M or ABT4Mmu in the absence (open box) or presence of theophylline (shaded box) is shown. The data represent mean ± SEM. * *P *< 0.04 versus mutant is significant.

To determine whether theophylline-induced sequestering of branchpoint can control splicing in living cells, we inserted the DNA that encodes ABT4M pre-mRNA into the mammalian expression vector pcDNA 3.1/ Myc-His(-) C yielding pcABT4M. A mutant (pcABT4Mmu) that does not bind to theophylline was also constructed. Next, HeLa cells were transfected transiently with pcABT4M, pcABT4Mmu or empty vector and treated with theophylline or buffer. After 24 h incubation, cells were harvested, and total RNA was isolated. Reverse transcription followed by PCR shown in Figure 7A demonstrates that theophylline can regulate the alternative splicing of ABT4M pre-mRNA. In contrast, theophylline had a less significant effect on the splicing of ABT4Mmu pre-mRNA, suggesting that binding of theophylline to its cognate RNA is necessary for controlling alternative splicing (Figure 7A, compare lanes 4 and 5 with lanes 6 and 7; also see Fig. 7B). We note that theophylline-dependent decrease in exon 2 included mRNA is not accompanied by a corresponding increase in exon 2 skipping. This suggests that although a block in the assembly of components involved in proximal 3' ss recognition prevented exon inclusion, the effect was not strong enough to influence a switch in splice site choice.

## Discussion

We have previously shown that insertion of 3' ss AG within a theophylline aptamer inhibits pre-mRNA splicing at the second step specifically by theophylline [28]. Since selection of BPS is made during early stages of the splicing pathway, we reasoned that sequestering of BPS within RNA-theophylline complex might be an effective strategy for controlling alternative splicing.

To test this hypothesis, we constructed a series of model pre-mRNAs in which BPS was engineered to be part of theophylline-binding aptamer (Fig. 1A). We found that these substrates underwent normal splicing, albeit with lower efficiency, when incubated with HeLa nuclear extract. Significantly, splicing of these substrates was inhibited specifically by theophylline (Fig. 1B). The observed low efficiency of splicing of these substrates could be due to a relatively long BPS-to-poly-(U) distance and/or the effect of purine residues that separate BPS from the poly-(Y) tract; both of these factors have been shown to affect lariat formation and exon ligation [46]. Indeed, relocation of BPS into lower aptamer stem, which decreased BPS-to-poly-(U) distance as well as excluded purine residues, improved splicing efficiency (compare the amount of lariat product and mRNA in Fig. 5B and Fig. 1B). In addition, unavailability of a competing BPS likely forced the splicing machinery to select a structured branchpoint, thus affecting the splicing efficiency. Support for this explanation comes from a previously reported study in which sequestering of a splice site within artificial hairpin sequence was found to be less detrimental to the splicing provided an alternative splice site is available [40].

Given the affinity (equilibrium dissociation constant or *K*_*d*_) of theophylline for cognate RNA aptamer is ~0.3 mM [29], an incomplete splicing inhibition at 2.0 mM theophylline is intriguing (Fig. 1B). We can offer the following explanations to this observation. First, the kinetic effect likely prevented RNA-theophylline complex from complete inhibition of splicing. It has been previously reported that the association kinetics of theophylline-aptamer binding is more than 1000-times slower than a diffusion controlled rate, which makes the conformational change in aptamer the rate limiting step for the formation of RNA-theophylline complex [47]. Thus, even though 2.0 mM theophylline is expected to saturate RNA aptamer, the slow association kinetics likely prevented complete splicing inhibition. Second, differential requirement of Mg^2+ ^for binding of theophylline to its cognate RNA and in vitro splicing may be the reason for incomplete splicing repression. Whereas efficient binding of theophylline to RNA aptamer requires 5.0 mM Mg^2+ ^[29,48], ~3.0 mM Mg^2+ ^has been found optimum for in vitro splicing [49]. Therefore, the residual splicing could be the result of a thermodynamically less stable RNA-theophylline-complex. Third, it is possible that theophylline also binds to other cellular factor(s), which may not affect splicing, but could decrease the amount of available theophylline. Nevertheless, theophylline dependent inhibition of splicing is highly specific (Fig. 2) and is carried out within the range of concentrations that has been reported in other studies. For example, An *et al *showed that 1–10 mM theophylline is required to affect the cleavage of an aptamer-fused shRNA by Dicer [50]. Similarly, translation repression of a CAT reporter containing theophylline-binding aptamer in wheat germ extracts required 1 mM theophylline [51].

Our data suggest that the size of lower theophylline aptamer stem as well as the location of BPS within aptamer affects the degree of splicing inhibition. For example, increasing the length of lower theophylline aptamer stem from a single to eight bp improved the efficiency of splicing inhibition by ~3-fold (Fig. 4B), suggesting a linear relationship between the length of the stem and the percent splicing inhibition. The insertion of BPS within lower theophylline aptamer stem although improved splicing efficiency, it also rendered pre-mRNA to be less responsive to theophylline-dependent splicing inhibition (cf. Fig. 4B, lane 5 and Fig. 5B lane 1). The most straightforward explanation to this observation is that when present in the aptamer lower stem, the accessibility of BPS to the spliceosome is energetically more favorable compared to its presence in the upper stem whose unwinding may require additional ~8.9 kcal/mole [52], the binding energy of theophylline to RNA aptamer.

Differential BPS recognition plays an important role in the regulation of alternative splicing [53,54]. For example, a splicing suppressor element that binds to SR proteins, 3RE, has been shown to regulate adenovirus major late IIIa pre-mRNA alternative splicing by preventing the binding of U2 snRNP to the BPS [55,56]. Here in this study we have shown that theophylline induced sequestering of BPS can influence alternative splicing both in vitro and in cultured cells (Fig. 6 and 7). Our data suggest that theophylline-mediated exon 2 skipping is due to intron 1 branchpoint sequestration, which allows intron 2 branchpoint to attack two alternative 5' splice sites for step I of the splicing. Two lines of evidence support this interpretation. First, splicing of ABT0M-6M pre-mRNAs yielded intron 1-retained mRNAs whose amount increased in the presence of theophylline (Fig. 6B, RNA moving just above 300 nt size marker). Second, theophylline shifted splicing in favor of exon 2 excluded mRNA by enabling intron 2 branchpoint to attack exon 1 5' ss. This effect is more pronounced in ABT4M where the strength of exon 2 5' ss is moderate; a very weak and a very strong exon 2 5' ss is expected to be skipped or included, respectively. An alternative explanation for theophylline-mediated exon 2 skipping is the process of exon definition. In this scenario sequestering of intron 1 branchpoint may interfere in the recognition of exon 2 3' ss, which in turn may compromise U1 snRNP binding to the downstream 5' ss resulting in exon skipping.

Quantitative analysis of data indicated that theophylline shifted exon 2 excluded/exon 2 included mRNA ratio between 2 to 3-fold in all of the tested substrates. It is important to point out that alternative splicing typically involves relatively small (< 10-fold) changes in isoform ratio, and the observed changes in the splice variant ratios may prove to be ideal for controlling the splicing of many biologically important pre-mRNAs. Although the present work represents only a proof-of-principle study, this system can be further fine-tuned to influence alternative splicing. For example, simultaneous sequestration of intron 1 branchpoint sequence and the 5' splice site of exon 2 has the potential to alter the splice variant ratio by several fold.

A previous report by Solnick and Lee analyzing the splicing pattern of artificial RNA secondary structure indicates that inverted repeat inhibits splicing in vitro, but failed to exert any effect in vivo unless the size of the stem is more than 50 nucleotides of perfect complementarity [57]. How, then, does theophylline induced secondary structure with a significantly short stem size compared with the one employed by these authors could influence splicing in vivo? This could be attributed to the intricate network of hydrogen bonds and stacking interactions that locks theophylline into the aptamer core thereby providing stability to the RNA secondary structure [35]. Since RNA secondary structure formed in Solnick and Lee study does not appear to be bound by a small molecule or a protein, the cellular RNA unwinding activity could have destabilized RNA secondary structure allowing normal splicing. Alternatively the use of larger loop size by Solnick and Lee could have allowed assembly of splicing factors on the nascent RNA while the other half of the inverted repeat is undergoing transcription [42].

## Conclusion

In conclusion, we have demonstrated that the RNA affinity of theophylline can be exploited to develop a system for conditional control of splicing. The modulation of mRNA splicing by theophylline offers several advantages. For example, theophylline-mediated control of splicing is flexible and can regulate splicing before or after the completion of the first step [28]. The fact that theophylline is a known drug with favorable pharmacokinetics and cellular uptake properties, this provides the possibility for controlling splicing of a *trans *gene in a gene therapy setting where the target gene expression could be controlled in a ligand-dependent manner.

## Methods

### Plasmid Construction

Plasmids encoding AdBPT12AG, 15AG and 18AG pre-mRNAs are derivatives of pAdMLPar [58] and were constructed following PCR-based cloning. In the first step, the sequence encompassing Hind III-Sal I region of pAdMLPar was replaced by the sequence (5'-AAGCTTTCGGGTGATACCAGTCAGCGTCTTGCTGACCCTTGGCAGCACCTTTTTTCAGGTCGAC-3') using PCR based mutagenesis with oligonucleotides #35745 (5'-CCTTGGCAGCACCTTTTTTCAGGTCGACGTTGAGGACAAAC-3') and 35744 (5'-GTCAGCAAGACGCTGACTGGTATCACCCGAAAGCTTCAAGGAAAC-3') as forward and reverse primers, respectively. The amplified DNA was then circularized using T4 DNA ligase to yield plasmid pAdBPT9AG. In the second step, PCR amplification of pAdBPT9AG using oligonucleotide T7 (5'-TAATACGACTCACTATAGGG-3') as forward primer and oligonucleotide #37457 (5'-AACGTCGACCTGAAAAAAAAAGGTGCTGCCAAGGGTCAG-3'), or oligonucleotide # 37458 (5'-AACGTCGACCTGAAAAAAAAAGAAGGTGCTGCCAAGGGTCAG-3') or #37459 (5'-AACGTCGACCTGAAAAAAAAAGAAAAAGGTGCTGCCAAGGGTCAG-3') as reverse primer yielded DNA fragments containing EcoR I and Sal I restriction sites. Finally, EcoR I and Sal I digested DNA fragments were introduced into pAdMLPar backbone to yield the desired constructs. Plasmids pMAdBPT15AG and pAdBPT15AG-1S were constructed by modified site-directed mutagenesis [59] consisting of two PCR amplification steps. In the first step, T7 oligonucleotide and the mutagenic primer #44300 (5'-TTCCTTGAAGCTTTCGGGTGGATGGTGTCAGCGTCTTGCTGACCCT-3') or oligonucleotide #37460 (5'-CAACGTCGACCTGAAAAAAAAAGCTGCCAAGGGTCAGCAAGACGCTGACTGGTATCGAAAGCTTCAAGGAAACCC-3') was used as forward and reverse primer, respectively. In the second PCR, the product of the first PCR served as the forward primer and SP6 (5'-ATTTAGGTGACACTATAGAA-3') as reverse primer. The construction of pAdBPT15AG-8S was carried out by PCR using pAdML21AG as template and oligonucleotide #38219(5'-TCTTGCTGACCCTTGGCAGCTCCTTCTTTTTTTTTCAGGTCGACG-3') and oligonucleotide #38221 (5'-CGCTGACTGGTATCTCCTTCTCGAAAGCTTCAAGGAAACCC-3') as forward and reverse primers, respectively. The resulting DNA fragments were circularized by T4 DNA ligase. To create pAdBPT15AG-LS, site-directed mutagenesis using T7 as forward primer, oligonucleotide SP6 as reverse primer and oligonucleotide #39393 (5'-TTGAAGCTTTCGGTCAGCAGATACCAGCATCGTCTTGATGCCCTTGGCAGCTGCTGACTTTTTTTCTTTTTTTTTCAGGT-3') as mutagenic primer was used. The resulting DNA fragment was digested with EcoR I and Xba I, and cloned into pSP72 vector.

To generate alternative splicing constructs, 3' half of pRG1 [44] was PCR amplified using oligonucleotide #39559 (5'-ATCGCGGATCCGTGGTGTGTCCTAGCATGTAGAACTGGTTACC-3'), or #39561 (5'-ATCGCGGATCCGTGGTGGGTCCTAGCATGTAGAACTGGTTACC-3'), or #39562 (5'-ATCGCGGATCCGTGGTTAGTCCTAGCATGTAGAACTGGTTACC-3'), or #39563 (5'-ATCGCGGATCCGAGGTTGGTCCTAGCATGTAGAACTGGTTACC-3') as forward primer and oligonucleotide #39368 (5'-TAGAGGATCCCCACTGGAAAGACCG-3') as reverse primer, respectively. The PCR amplified DNA was subjected to BamH I digestion followed by subcloning into pAdBPT15AG to yield plasmids pABT0M-6M. In vivo splicing constructs (pcABT0M-6M) were generated by subcloning the PCR amplified DNA encoding pre-mRNAs ABT0M-6M into Nhe I and Xba I digested pcDNA 3.1/ Myc-His(-) C The oligonucleotide #40550 (5'-ATAGGGAGACCGGCGCTAGCCCGCATCGCTGTCTGCGAGGG-3') and SP6 were used as forward and reverse primers, respectively. The construction of pcABT4Mmu was carried out following site-directed mutagenesis with oligonucleotide T7 as forward primer, oligonucleotide #46436 (5'-CCTTGAAGCTTTCGAGAAGGAGGATGGTGTCAGCGTCTTGCTGACCCTTGG-3') as mutagenic primer, and oligonucleotide #44759 (5'-TCAATGATGATGATGATGATGG-3') as reverse primer and pcABT4M as template. The PCR amplified DNA was subcloned into Nhe I and EcoR I digested pcDNA 3.1/ Myc-His(-) C vector.

### Cell culture and transfection

HeLa cells were grown in Dulbecco's modified Eagle medium (Cellgro, Herndon, VA) supplemented with L-glutamine and 10% (v/v) fetal bovine serum (Omega scientific, Tarzana, CA). Cells were maintained under standard incubation conditions (humidified atmosphere, 95% air, 5% CO_2_, 37°C). For transfection, 4.5 × 10^5 ^cells were seeded in a 6-well plate a day prior to transfection. Cells were grown to approximately 60–70% confluencey and transfected with 1 μg of DNA by using Lipofectamine 2000 (Invitrogen) according to the manufacturer's protocol except that prior to the addition of DNA-Lipofectamine 2000 complex, the media was replaced by fresh media containing indicated concentration of theophylline (≥ 99.0%, Fluka). A freshly prepared solution of theophylline (25 mM stock solution in sterile water) was used.

### RNA Isolation and RT-PCR

Total RNA was prepared using RNeasy mini kit (Qiagen) and treated with DNase I (Roche) following the manufacturer's protocol. Five microgram RNA was subjected to reverse transcription using oligo (dT) and MMLV reverse transcriptase (Invitrogen) essentially according to the manufacturer's instructions. Next, 1 μl of RT mixture was PCR amplified using oligonucleotide #43573 (5'-GGGCCAGCTGTTGGGGTCGA-3') and #43754 (5'-GGGCCAGCTGTTGGGCTCGC-3') as forward primer and oligonucleotide #39368 as common reverse primer. The PCR products were analyzed by electrophoresis on 8% polyacrylamide or 2.5% agarose gel, and quantified by ImageJ software version 1.36b (National Institutes of Health).

### In vitro transcription

Linearized plasmid (1 μg) was used as template for run-off transcription [60]. A typical (12.5-μL) in vitro transcription reaction consisted of 40 mM Tris-HCl (pH 8.0), 2.0 mM spermidine, 10 mM DTT, 20 mM MgCl_2_, NTP mixture (0.4 mM CTP and ATP, and 0.1 mM GTP and UTP), 2.0 mM cap analog (NEB), ~10 μCi [∝-^32^P] UTP, 10–20 units T7 polymerase (NEB). After incubation at 37°C for 2 h, the reaction was terminated by adding 12.5 μL stop buffer and RNA was purified on a 8% denaturing polyacrylamide gel.

### In vitro splicing

HeLa-S3 cells for nuclear extracts were purchased from the National Cell Culture Center (Cellex Biosciences, Minneapolis, MN) received as cell pellet on wet ice. Nuclear extracts were prepared as described [61]. Splicing assays were performed in 12.5-μL reaction mixtures containing 50% nuclear extract. To ensure that theophylline binds to its RNA target, a solution consisting of ^32^P-labeled pre-mRNA (5–10 fmol, ~10,000 cpm per reaction), indicated concentration of theophylline, 0.5 μl BC300 (20 mM HEPES, pH 8.0, 20% glycerol, 300 mM KCl, 0.2 mM EDTA) and 0.25 μl 160 mM MgCl_2 _was heated at 65°C for 5 min, followed by 20 min incubation at room temperature. Next, 0.5 mM ATP, 20 mM creatine phosphate, 0.4 units of RNasin (Promega), 1.0 mM DTT, 6.25 μl HeLa nuclear extract, and water up to 12.5 μl (all concentrations are final) was added and incubation continued at 30°C for the indicated time. Where indicated, theophylline was substituted by caffeine or water. To terminate the splicing reaction, 87.5 μl proteinase K buffer consisting of 25 μg proteinase K (Roche), 100 mM Tris-HCl, pH 7.5, 12.5 mM EDTA, pH 8.0, 150 mM NaCl and 1% SDS was added and incubated at 55°C for 15 min. After phenol-chloroform extraction, the extracted RNAs were resolved by electrophoresis on 13% (19:1) 7M-urea polyacrylamide gel (or as indicated in the figure legends), visualized and quantified using a Molecular Dynamics PhosphorImager and the ImageQuant version 4.2 software.

### Branchpoint mapping

Branchpoint mapping was performed essentially according to the published protocol [34]. Preparative scale (100 μl) splicing reactions were performed as described earlier and lariat intermediates were visualized by autoradiography. After gel elution, one half of the lariat intermediate was subjected to debranching reaction with S100 extract. The branchpoint was mapped by comparing the primer extension products of the lariat intermediate and S100 treated lariat intermediate. In brief, 10–15 pmol R5 primer (5'-ATGCCTGCAGGTCGACTCTAGAGG-3') was 5' end labeled with [ϒ-^32^P]ATP by incubating at 37°C in the presence of 20 U of T4 polynucleotide kinase (NEB) and 1× kinase buffer (NEB). Unincorporated [ϒ-^32^P]ATP was removed by using MicroSpin G-25 Sephadex column (GE Healthcare). Intact or debranched lariat RNA and ~1 pmole labeled primer were mixed in 20 μl of high salt buffer containing 40 mM PIPES (pH 6.4), 1 mM EDTA, 400 mM NaCl and 0.2% SDS. Primer was annealed to lariat intermediate by heating at 95°C for 1 min and slow cooling to room temperature over a period of 30–60 min. Reaction mixture was extracted with phenol:chloroform followed by ethanol precipitation. The RNA-primer hybrid was resuspended in 10 μl reaction buffer containing 50 mM Tris (pH 8.0), 74 mM KCl, 6 mM MgCl_2_, 10 mM DTT, 1 mM each dNTPs, 1μl of AMV reverse-transcriptase (Invitrogen) and incubated for 1 h at 42°C. Samples were treated with 1 μl of DNase free RNase I (NEB) for 40 min at 37°C. Next, reaction mixture was extracted with phenol:chloroform, ethanol precipitated, and resuspended in loading buffer. A sequencing ladder was generated using a Sequnase version 2.0 DNA sequencing kit, the parental plasmid and the R5 primer. The products were analyzed on a 13% polyacrylamide denaturing gel.

## Abbreviations

BPS, Branchpoint sequence; bp, base-pair; nt, nucleotide; SS, splice site; TBA, theophylline-binding aptamer

## Competing interests

The author(s) declare that they have no competing interests.

## Authors' contributions

DSK performed splicing experiments. VG constructed AdT+10 template and carried out in vitro splicing experiment. KJD participated in splicing experiments with caffeine. RKG provided overall direction to the project and prepared the manuscript. All authors read and approved the final draft of the manuscript.

## Supplementary Material

Additional File 1 **Annotated sequences of the reporter constructs**. Additional file 1 is a PDF file presenting the annotated sequences of the reporter constructs used in this study. Slashes indicate 5' and 3' splice sites, A denotes branchpoint nucleotide, and underlined sequence represents theophylline aptamer or mutant. The distal 5' splice site in ABT0M-6M and ABT4Mmu pre-mRNAs is shown in bold letters. Click here for file

Additional File 2 **The RNA secondary structure of MAdBPT15AG**. Additional file 2 is a TIF file illustrating the RNA secondary structure of MAdBPT15AG drawn by using Zucker's M-FOLD program [62]. Click here for file

## References

[B1] Konarska MM, Query CC (2005). Insights into the mechanisms of splicing: more lessons from the ribosome. Genes Dev.

[B2] Butcher SE, Brow DA (2005). Towards understanding the catalytic core structure of the spliceosome. Biochem Soc Trans.

[B3] Nilsen TW (2003). The spliceosome: the most complex macromolecular machine in the cell?. Bioessays.

[B4] Blencowe BJ (2006). Alternative splicing: new insights from global analyses. Cell.

[B5] Singh R, Valcarcel J (2005). Building specificity with nonspecific RNA-binding proteins. Nat Struct Mol Biol.

[B6] Black DL (2003). Mechanisms of alternative pre-messenger RNA splicing. Annu Rev Biochem.

[B7] Graveley BR (2001). Alternative splicing: increasing diversity in the proteomic world. Trends Genet.

[B8] Stetefeld J, Ruegg MA (2005). Structural and functional diversity generated by alternative mRNA splicing. Trends Biochem Sci.

[B9] Maniatis T, Tasic B (2002). Alternative pre-mRNA splicing and proteome expansion in metazoans. Nature.

[B10] Mironov AA, Fickett JW, Gelfand MS (1999). Frequent alternative splicing of human genes. Genome Res.

[B11] Johnson JM, Castle J, Garrett-Engele P, Kan Z, Loerch PM, Armour CD, Santos R, Schadt EE, Stoughton R, Shoemaker DD (2003). Genome-wide survey of human alternative pre-mRNA splicing with exon junction microarrays. Science.

[B12] Kan Z, Rouchka EC, Gish WR, States DJ (2001). Gene structure prediction and alternative splicing analysis using genomically aligned ESTs. Genome Res.

[B13] Modrek B, Resch A, Grasso C, Lee C (2001). Genome-wide detection of alternative splicing in expressed sequences of human genes. Nucleic Acids Res.

[B14] Garcia-Blanco MA (2006). Alternative splicing: therapeutic target and tool. Prog Mol Subcell Biol.

[B15] Faustino NA, Cooper TA (2003). Pre-mRNA splicing and human disease. Genes Dev.

[B16] Dredge BK, Polydorides AD, Darnell RB (2001). The splice of life: alternative splicing and neurological disease. Nat Rev Neurosci.

[B17] Gaur RK (2006). RNA interference: a potential therapeutic tool for silencing splice isoforms linked to human diseases. Biotechniques.

[B18] Steinman HA, Burstein E, Lengner C, Gosselin J, Pihan G, Duckett CS, Jones SN (2004). An alternative splice form of Mdm2 induces p53-independent cell growth and tumorigenesis. J Biol Chem.

[B19] Xerri L, Parc P, Brousset P, Schlaifer D, Hassoun J, Reed JC, Krajewski S, Birnbaum D (1996). Predominant expression of the long isoform of Bcl-x (Bcl-xL) in human lymphomas. Br J Haematol.

[B20] Venables JP (2004). Aberrant and alternative splicing in cancer. Cancer Res.

[B21] Dominski Z, Kole R (1993). Restoration of correct splicing in thalassemic pre-mRNA by antisense oligonucleotides. Proc Natl Acad Sci USA.

[B22] Heidenreich O, Kang SH, Xu X, Nerenberg M (1995). Application of antisense technology to therapeutics. Mol Med Today.

[B23] Wilusz JE, Devanney SC, Caputi M (2005). Chimeric peptide nucleic acid compounds modulate splicing of the bcl-x gene in vitro and in vivo. Nucleic Acids Res.

[B24] Villemaire J, Dion I, Elela SA, Chabot B (2003). Reprogramming alternative pre-messenger RNA splicing through the use of protein-binding antisense oligonucleotides. J Biol Chem.

[B25] Cartegni L, Krainer AR (2003). Correction of disease-associated exon skipping by synthetic exon-specific activators. Nat Struct Biol.

[B26] Lopez AJ (1998). Alternative splicing of pre-mRNA: developmental consequences and mechanisms of regulation. Annu Rev Genet.

[B27] Graveley BR (2005). Small molecule control of pre-mRNA splicing. RNA.

[B28] Kim DS, Gusti V, Pillai SG, Gaur RK (2005). An artificial riboswitch for controlling pre-mRNA splicing. RNA.

[B29] Zimmermann GR, Wick CL, Shields TP, Jenison RD, Pardi A (2000). Molecular interactions and metal binding in the theophylline-binding core of an RNA aptamer. RNA.

[B30] Zimmermann GR, Shields TP, Jenison RD, Wick CL, Pardi A (1998). A semiconserved residue inhibits complex formation by stabilizing interactions in the free state of a theophylline-binding RNA. Biochemistry.

[B31] Ruskin B, Greene JM, Green MR (1985). Cryptic branch point activation allows accurate in vitro splicing of human beta-globin intron mutants. Cell.

[B32] Reed R, Maniatis T (1985). Intron sequences involved in lariat formation during pre-mRNA splicing. Cell.

[B33] Padgett R, Konarska M, Aebi M, Hornig H, Weissmann C, Sharp P (1985). Nonconsensus branch-site sequences in the in vitro splicing of transcripts of mutant rabbit beta-globin genes. Proc Natl Acad Sci USA.

[B34] Ruskin B, Green MR (1990). RNA lariat debranching enzyme as tool for analyzing RNA structure. Methods Enzymol.

[B35] Zimmermann GR, Jenison RD, Wick CL, Simorre JP, Pardi A (1997). Interlocking structural motifs mediate molecular discrimination by a theophylline-binding RNA. Nat Struct Biol.

[B36] Howe KJ, Ares M (1997). Intron self-complementarity enforces exon inclusion in a yeast pre-mRNA. Proc Natl Acad Sci USA.

[B37] Lian Y, Garner HR (2005). Evidence for the regulation of alternative splicing via complementary DNA sequence repeats. Bioinformatics.

[B38] Buratti E, Baralle FE (2004). Influence of RNA secondary structure on the pre-mRNA splicing process. Mol Cell Biol.

[B39] Deshler JO, Rossi JJ (1991). Unexpected point mutations activate cryptic 3' splice sites by perturbing a natural secondary structure within a yeast intron. Genes Dev.

[B40] Goguel V, Wang Y, Rosbash M (1993). Short artificial hairpins sequester splicing signals and inhibit yeast pre-mRNA splicing. Mol Cell Biol.

[B41] Solnick D (1985). Alternative splicing caused by RNA secondary structure. Cell.

[B42] Eperon LP, Graham IR, Griffiths AD, Eperon IC (1988). Effects of RNA secondary structure on alternative splicing of pre-mRNA: is folding limited to a region behind the transcribing RNA polymerase?. Cell.

[B43] Goodall GJ, Filipowicz W (1991). Different effects of intron nucleotide composition and secondary structure on pre-mRNA splicing in monocot and dicot plants. EMBO J.

[B44] Gaur RK, McLaughlin LW, Green MR (1997). Functional group substitutions of the branchpoint adenosine in a nuclear pre-mRNA and a group II intron. RNA.

[B45] Roca X, Sachidanandam R, Krainer AR (2005). Determinants of the inherent strength of human 5' splice sites. RNA.

[B46] Reed R (1989). The organization of 3' splice-site sequences in mammalian introns. Genes Dev.

[B47] Jucker FM, Phillips RM, McCallum SA, Pardi A (2003). Role of a heterogeneous free state in the formation of a specific RNA-theophylline complex. Biochemistry.

[B48] Jenison RD, Gill SC, Pardi A, Polisky B (1994). High-resolution molecular discrimination by RNA. Science.

[B49] Krainer AR, Maniatis T, Ruskin B, Green MR (1984). Normal and mutant human beta-globin pre-mRNAs are faithfully and efficiently spliced in vitro. Cell.

[B50] An CI, Trinh VB, Yokobayashi Y (2006). Artificial control of gene expression in mammalian cells by modulating RNA interference through aptamer-small molecule interaction. RNA.

[B51] Harvey I, Garneau P, Pelletier J (2002). Inhibition of translation by RNA-small molecule interactions. RNA.

[B52] Gouda H, Kuntz ID, Case DA, Kollman PA (2003). Free energy calculations for theophylline binding to an RNA aptamer: Comparison of MM-PBSA and thermodynamic integration methods. Biopolymers.

[B53] Hovhannisyan RH, Warzecha CC, Carstens RP (2006). Characterization of sequences and mechanisms through which ISE/ISS-3 regulates FGFR2 splicing. Nucleic Acids Res.

[B54] Kralovicova J, Houngninou-Molango S, Kramer A, Vorechovsky I (2004). Branch site haplotypes that control alternative splicing. Hum Mol Genet.

[B55] Dauksaite V, Akusjarvi G (2002). Human splicing factor ASF/SF2 encodes for a repressor domain required for its inhibitory activity on pre-mRNA splicing. J Biol Chem.

[B56] Kanopka A, Muhlemann O, Akusjarvi G (1996). Inhibition by SR proteins of splicing of a regulated adenovirus pre-mRNA. Nature.

[B57] Solnick D, Lee SI (1987). Amount of RNA secondary structure required to induce an alternative splice. Mol Cell Biol.

[B58] Gozani O, Patton JG, Reed R (1994). A novel set of spliceosome-associated proteins and the essential splicing factor PSF bind stably to pre-mRNA prior to catalytic step II of the splicing reaction. EMBO J.

[B59] Wu W, Jia Z, Liu P, Xie Z, Wei Q (2005). A novel PCR strategy for high-efficiency, automated site-directed mutagenesis. Nucleic Acids Res.

[B60] Gaur RK, Krupp G (1993). Enzymatic RNA synthesis with deoxynucleoside 5'-O-(1-thiotriphosphates). FEBS Lett.

[B61] Dignam JD, Lebovitz RM, Roeder RG (1983). Accurate transcription initiation by RNA polymerase II in a soluble extract from isolated mammalian nuclei. Nucleic Acids Res.

[B62] mfold Web Server. http://frontend.bioinfo.rpi.edu/zukerm/home.html.

