# Supplementary material for: Ligand-induced sequestering of branchpoint sequence allows conditional control of splicing
Source: BMC Mol Biol. 2008 Feb 12;9:23. doi: 10.1186/1471-2199-9-23 (PMC2275289; doi:10.1186/1471-2199-9-23)
Supplement: Additional File 1 — Annotated sequences of the reporter constructs. Additional file 1 is a PDF file presenting the annotated sequences of the reporter constructs used in this study. Slashes indicate 5' and 3' splice sites, A denotes branchpoint nucleotide, and underlined sequence represents theophylline aptamer or mutant. The distal 5' splice site in ABT0M-6M and ABT4Mmu pre-mRNAs is shown in bold letters. [file 1471-2199-9-23-S1.pdf]

The pre-mRNA sequences encoded by plasmids used in this study are shown. Slashes indicate 5′ and 3′ splice sites, A denotes branchpoint nucleotide, and underlined sequence represents theophylline aptamer or mutant. The distal 5′ splice site in ABT0-6M and ABT4mu pre-mRNAs is shown in bold letters.

AdBPT12AG:

5′-
GGGAGACCGGCAGAUCAGCUUGGCCGCGUCCAUCUGGUCAUCUAGGAUCUGAUAU
CAUCGAUGAAUUCGAGCUCGGUACCCCGUUCGUCCUCACUCUCUUCCGCAUCGCU
GUCUGCGAGGGCCAGCUGUUGGG/GUGAGUACUCCCUCUCAAAAGCGGGCAUGAC
UUCUGCCCUCGAGUUAUUAACCCUCACUAAAGGCAGUAGUCAAGGGUUUCCUUGA
AGCUUUCGGGUGAUACCAGUCAGCGUCUUGCUGACCCUUGGCAGCACCUUUUUUU
UUCAG/GUCGACGUUGAGGACAAACUCUUCGCGGUCUUUCCAGUACUCUUGGAUC
CUCUAGA-3′.

AdBPT15AG:

5′-
GGGAGACCGGCAGAUCAGCUUGGCCGCGUCCAUCUGGUCAUCUAGGAUCUGAUAU
CAUCGAUGAAUUCGAGCUCGGUACCCCGUUCGUCCUCACUCUCUUCCGCAUCGCU
GUCUGCGAGGGCCAGCUGUUGGG/GUGAGUACUCCCUCUCAAAAGCGGGCAUGAC
UUCUGCCCUCGAGUUAUUAACCCUCACUAAAGGCAGUAGUCAAGGGUUUCCUUGA
AGCUUUCGGGUGAUACCAGUCAGCGUCUUGCUGACCCUUGGCAGCACCUUCUUUU
UUUUUCAG/GUCGACGUUGAGGACAAACUCUUCGCGGUCUUUCCAGUACUCUUGG
AUCCUCUAGA-3′.

AdBPT18AG:

5′-
GGGAGACCGGCAGAUCAGCUUGGCCGCGUCCAUCUGGUCAUCUAGGAUCUGAUAU
CAUCGAUGAAUUCGAGCUCGGUACCCCGUUCGUCCUCACUCUCUUCCGCAUCGCU
GUCUGCGAGGGCCAGCUGUUGGG/GUGAGUACUCCCUCUCAAAAGCGGGCAUGAC
UUCUGCCCUCGAGUUAUUAACCCUCACUAAAGGCAGUAGUCAAGGGUUUCCUUGA
AGCUUUCGGGUGAUACCAGUCAGCGUCUUGCUGACCCUUGGCAGCACCUUUUUCU
UUUUUUUUCAG/GUCGACGUUGAGGACAAACUCUUCGCGGUCUUUCCAGUACUCU
UGGAUCCUCUAGA-3′.

MAdBPT15AG:

5′-
GGGAGACCGGCAGAUCAGCUUGGCCGCGUCCAUCUGGUCAUCUAGGAUCUGAUAU
CAUCGAUGAAUUCGAGCUCGGUACCCCGUUCGUCCUCACUCUCUUCCGCAUCGCU
GUCUGCGAGGGCCAGCUGUUGGG/GUGAGUACUCCCUCUCAAAAGCGGGCAUGAC
UUCUGCCCUCGAGUUAUUAACCCUCACUAAAGGCAGUAGUCAAGGGUUUCCUUGA
AGCUUUCGGGUGGAUGGUGUCAGCGUCUUGCUGACCCUUGGCAGCACCUUCUUU
UUUUUCAG/GUCGACGUUGAGGACAAACUCUUCGCGGUCUUUCCAGUACUCUUG
GAUCCUCUAGA-3′.

AdT+10
GGGAGACCGGCAGAUCAGCUUGGCCGCGUCCAUCUGGUCAUCUAGGAUCUGAUAU
CAUCGAUGAAUUCGAGCUCGGUACCCCGUUCGUCCUCACUCUCUUCCGCAUCGCU
GUCUGCGAGGGCCAGCUGUUGGG/GUGAGUACUCGGUGAUACCAGCCGAAAGGCC
CUUGGCAGCACCCCUCUCAAAAGCGGGCAUGACUUCUGCCCUCGAGUUAUUAACC
CUCACUAAAGGCAGUAGUCAAGGGUUUCCUUGAAGCUUUCGUGCUGACUUUUUU
UCUUUUUUUUUCAG/GUCGACGUUGAGGACAAACUCUUCGCGGUCUUUCCAGUAC
UCUUGGAUCCUCUAGAGUCGAGUUCUAUAGUGUCACCUAAAU

AdBPT15AG-1S:
5′-
GGGAGACCGGCAGAUCAGCUUGGCCGCGUCCAUCUGGUCAUCUAGGAUCUGAUAU
CAUCGAUGAAUUCGAGCUCGGUACCCCGUUCGUCCUCACUCUCUUCCGCAUCGCU
GUCUGCGAGGGCCAGCUGUUGGG/GUGAGUACUCCCUCUCAAAAGCGGGCAUGAC
UUCUGCCCUCGAGUUAUUAACCCUCACUAAAGGCAGUAGUCAAGGGUUUCCUUGA
AGCUUUCGAUACCAGUCAGCGUCUUGCUGACCCUUGGCAGCUUUUUUUUUCAG/G
UCGACGUUGAGGACAAACUCUUCGCGGUCUUUCCAGUACUCUUGGAUCCUCUAGA
-3′.

AdBPT15AG-8S:
5′-
GGGAGACCGGCAGAUCAGCUUGGCCGCGUCCAUCUGGUCAUCUAGGAUCUGAUAU
CAUCGAUGAAUUCGAGCUCGGUACCCCGUUCGUCCUCACUCUCUUCCGCAUCGCU
GUCUGCGAGGGCCAGCUGUUGGG/GUGAGUACUCCCUCUCAAAAGCGGGCAUGAC
UUCUGCCCUCGAGUUAUUAACCCUCACUAAAGGCAGUAGUCAAGGGUUUCCUUGA
AGCUUUCGAGAAGGAGAUACCAGUCAGCGUCUUGCUGACCCUUGGCAGCUCCUUC
UUUUUUUUUCAG/GUCGACGUUGAGGACAAACUCUUCGCGGUCUUUCCAGUACUC
UUGGAUCCUCUAGA-3′.

AdBPT15AG-LS:
5′-
GGGAGACCGGCAGAUCAGCUUGGCCGCGUCCAUCUGGUCAUCUAGGAUCUGAUAU
CAUCGAUGAAUUCGAGCUCGGUACCCCGUUCGUCCUCACUCUCUUCCGCAUCGCU
GUCUGCGAGGGCCAGCUGUUGGG/GUGAGUACUCCCUCUCAAAAGCGGGCAUGAC
UUCUGCCCUCGAGUUAUUAACCCUCACUAAAGGCAGUAGUCAAGGGUUUCCUUGA
AGCUUUCGGUCAGCAGAUACCAGCAUCGUCUUGAUGCCCUUGGCAGCUGCUGACU
UCUUUUUUUUUCAG/GUCGACGUUGAGGACAAACUCUUCGCGGUCUUUCCAGUAC
UCUUGGAUCCUCUAGA-3′.

ABT0M:
5′-
GGGAGACCCAAGCUGGCUAGCCCGCAUCGCUGUCUGCGAGGGCCAGCUGUUGGG/
GUGAGUACUCCCUCUCAAAAGCGGGCAUGACUUCUGCCCUCGAGUUAUUAACCCU
CACUAAAGGCAGUAGUCAAGGGUUUCCUUGAAGCUUUCGAGAAGGAGAUACCAG
UCAGCGUCUUGCUGACCCUUGGCAGCUCCUUCUUUUUUUUUCAG/GUCGACGUUG
AGGACAAACUCUUCGCGGUCUUUCCAGUACUCUUGGAUCCGUG/**GUGUGU**CCUAG
CAUGUAGAACUGGUUACCUGCAGCCCAAGCUUGCUGCACGUCUAGGGCGCAGUAG
UCCAGGAUGUUUCCUUGAUGAUGGGCUCUGCUGACGCUGUCCCUUUUUUUUCCAC
AG/CUCGCGGUUGAGGACAAACUCUUCGCGGUCUUUCCAGUGGGGAUCCUCUA-3′.

ABT2M:
5′-
GGGAGACCCAAGCUGGCUAGCCCGCAUCGCUGUCUGCGAGGGCCAGCUGUUGGG/
GUGAGUACUCCCUCUCAAAAGCGGGCAUGACUUCUGCCCUCGAGUUAUUAACCCU
CACUAAAGGCAGUAGUCAAGGGUUUCCUUGAAGCUUUCGAGAAGGAGAUACCAG
UCAGCGUCUUGCUGACCCUUGGCAGCUCCUUCUUUUUUUUUCAG/GUCGACGUUG
AGGACAAACUCUUCGCGGUCUUUCCAGUACUCUUGGAUCCGUG/**GUGGGU**CCUAG
CAUGUAGAACUGGUUACCUGCAGCCCAAGCUUGCUGCACGUCUAGGGCGCAGUAG
UCCAGGAUGUUUCCUUGAUGAUGGGCUCUGCUGACGCUGUCCCUUUUUUUUCCAC
AG/CUCGCGGUUGAGGACAAACUCUUCGCGGUCUUUCCAGUGGGGAUCCUCUA-3′.

ABT4M:
5′-
GGGAGACCCAAGCUGGCUAGCCCGCAUCGCUGUCUGCGAGGGCCAGCUGUUGGG/
GUGAGUACUCCCUCUCAAAAGCGGGCAUGACUUCUGCCCUCGAGUUAUUAACCCU
CACUAAAGGCAGUAGUCAAGGGUUUCCUUGAAGCUUUCGAGAAGGAGAUACCAG
UCAGCGUCUUGCUGACCCUUGGCAGCUCCUUCUUUUUUUUUCAG/GUCGACGUUG
AGGACAAACUCUUCGCGGUCUUUCCAGUACUCUUGGAUCCGUG/**GUUAGU**CCUAG
CAUGUAGAACUGGUUACCUGCAGCCCAAGCUUGCUGCACGUCUAGGGCGCAGUAG
UCCAGGAUGUUUCCUUGAUGAUGGGCUCUGCUGACGCUGUCCCUUUUUUUUCCAC
AG/CUCGCGGUUGAGGACAAACUCUUCGCGGUCUUUCCAGUGGGGAUCCUCUA-3′.

ABT6M:
5′-
GGGAGACCCAAGCUGGCUAGCCCGCAUCGCUGUCUGCGAGGGCCAGCUGUUGGG/
GUGAGUACUCCCUCUCAAAAGCGGGCAUGACUUCUGCCCUCGAGUUAUUAACCCU
CACUAAAGGCAGUAGUCAAGGGUUUCCUUGAAGCUUUCGAGAAGGAGAUACCAG
UCAGCGUCUUGCUGACCCUUGGCAGCUCCUUCUUUUUUUUUCAG/GUCGACGUUG
AGGACAAACUCUUCGCGGUCUUUCCAGUACUCUUGGAUCCGAG/**GUUGGU**CCUAG
CAUGUAGAACUGGUUACCUGCAGCCCAAGCUUGCUGCACGUCUAGGGCGCAGUAG
UCCAGGAUGUUUCCUUGAUGAUGGGCUCUGCUGACGCUGUCCCUUUUUUUUCCAC
AG/CUCGCGGUUGAGGACAAACUCUUCGCGGUCUUUCCAGUGGGGAUCCUCUA-3′.

ABT4Mmu:
5′-
GGGAGACCCAAGCUGGCUAGCCCGCAUCGCUGUCUGCGAGGGCCAGCUGUUGGG/
GUGAGUACUCCCUCUCAAAAGCGGGCAUGACUUCUGCCCUCGAGUUAUUAACCCU
CACUAAAGGCAGUAGUCAAGGGUUUCCUUGAAGCUUUCGAGAAGGAGGAUGGUG
UCAGCGUCUUGCUGACCCUUGGCAGCUCCUUCUUUUUUUUUCAG/GUCGACGUUG
AGGACAAACUCUUCGCGGUCUUUCCAGUACUCUUGGAUCCGUG/**GUUAGU**CCUAG
CAUGUAGAACUGGUUACCUGCAGCCCAAGCUUGCUGCACGUCUAGGGCGCAGUAG
UCCAGGAUGUUUCCUUGAUGAUGGGCUCUGCUGACGCUGUCCCUUUUUUUUCCAC
AG/CUCGCGGUUGAGGACAAACUCUUCGCGGUCUUUCCAGUGGGGAUCCUCUA-3′.
