# Supplementary figures and images for: Ligand-induced sequestering of branchpoint sequence allows conditional control of splicing
Source: BMC Mol Biol. 2008 Feb 12;9:23. doi: 10.1186/1471-2199-9-23 (PMC2275289; doi:10.1186/1471-2199-9-23)

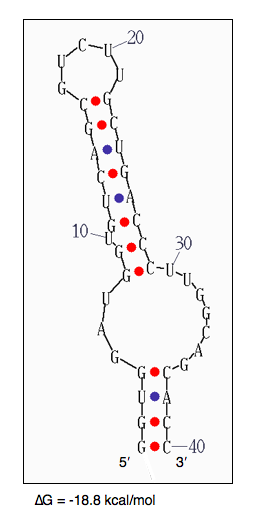

Supplement: Additional File 2 — The RNA secondary structure of MAdBPT15AG. Additional file 2 is a TIF file illustrating the RNA secondary structure of MAdBPT15AG drawn by using Zucker's M-FOLD program [62]. [file 1471-2199-9-23-S2.TIFF]
